# Supplementary material for: Distinctly Different Morphologies of Bimetallic Au-Ag Nanostructures and Their Application in Submicromolar SERS-Detection of Free Base Porphyrin
Source: Nanomaterials (Basel). 2021 Aug 26;11(9):2185. doi: 10.3390/nano11092185 (PMC8464754; doi:10.3390/nano11092185)
Supplement: Supplementary file 1 [file nanomaterials-11-02185-s001.zip › nanomaterials-1360441-supplementary.pdf]

Supporting Information

Distinctly Different Morphologies of Bimetallic Au-Ag Nanostructures and Their Application in Submicromolar SERS-Detection of Free Base Porphyrin

Iveta Vilímová and Karolína Šišková *

Department of Experimental Physics, Faculty of Science, Palacký University in Olomouc, Tř. 17. Listopadu 1192/12, 77146 Olomouc, Czech Republic; vilimovai4@gmail.com
* Correspondence: karolina.siskova@upol.cz

Calculation of monolayer Ag(I) coverage on Au seed (20 nm in diameter) surfaces:

Premises: The whole amount of Au(III) is reduced into AuNPs.

All Au nanoparticles (NPs) are spherical of mean diameter of 20 nm.

The density of bulk Au is considered.

Atomic radius of Ag as in bulk metal and spherical shape of Ag atom are taken into account.

All Ag(I) from $AgNO_3$ can be deposited atom by atom on each type of AuNP surface.

1) The amount of Au (mol) in a spherical Au NPs of 20 nm in diameter…**n(Au-in-NP)**:

$$R = 10\ \text{nm} = 1 \times 10^{-6}\ \text{cm} \tag{S1}$$

$$\rho(\text{Au}) = 19.30\ \text{g.cm}^{-3} \tag{S2}$$

$$\text{Ar(Au)} = 196.97 \tag{S3}$$

$$V_{sphere} = 4/3\ \pi\ R^3 \tag{S4}$$

$$\text{m(1AuNP)} = \rho(\text{Au}) * V_{sphere} = 19.30 * 4/3 * \pi * 10^{-18}\ \text{g} = 80.84 \times 10^{-18}\ \text{g} \tag{S5}$$

$$\text{n(Au-in-NP)} = \text{m(1AuNP)} / \text{Ar(Au)} = 80.84 \times 10^{-18} / 196.97\ \text{mol} = 4.10 \times 10^{-19}\ \text{mol} \tag{S6}$$

2) The concentration of Au(III) in AuBhr and AuCitr are as follows:

$$\text{c(Au-in-AuBhr)} = 2.2 * 9/84\ \text{mM} = 2.36 \times 10^{-4}\ \text{M} \tag{S7}$$

$$\text{c(Au-in-AuCitr)} = 2.48 \times 10^{-4}\ \text{M} \tag{S8}$$

3) How many Au particles are in the added Au seed solutions…**N(Au seeds)**?

0.5 mL of AuNPs serving as seeds is used in each syntheses

$$\text{N(Au seeds)} = \text{n(Au-in-AuBhr)} / \text{n(Au-in-NP)} \tag{S9}$$

Where n(Au-in-AuBhr) = c(Au-in-AuBhr) * V_{seeds} = $2.36 \times 10^{-4} * 0.5 \times 10^{-3}$ mol = 1.18×10^{-7} mol

$$\text{N(Au seeds)} = 1.18 \times 10^{-7} / 4.10 \times 10^{-19} = 2.87 \times 10^{11} \tag{S10}$$

There are theoretically 2.87×10^{11} Au particles which are entering the second step of seed-mediated growth procedure.

4) Area available on all Au particles serving as seeds…**A(allAuseeds):**

$$A = 4\ \pi\ R^2 \tag{S11}$$

$$R = 10\ \text{nm} \tag{S12}$$

$$A(allAuseeds) = N(Au\ seeds) * S = 2.87 \times 10^{11} * 4 * \pi * 100\ nm^2 = 3.61 \times 10^{14}\ nm^2 \quad (S13)$$

5) Area of 1 Ag atom…**A(Ag):**

$$A(Ag) = \pi\ r_{Ag}^2 \quad (S14)$$

$$r_{Ag} = 144\ pm = 0.144\ nm \quad (S15)$$

$$A(Ag) = 6.51 \times 10^{-2}\ nm^2 \quad (S16)$$

6) If monolayer coverage (MNLC) of Au seed surface by Ag atoms assumed, then how many mol of Ag atoms should be available in reaction solution?... **n(AgMNLC)**

$$N(AgMNLC) = A(allAuseeds) / A(Ag) = 3.61 \times 10^{14} / 6.51 \times 10^{-2} = 5.54 \times 10^{15} \quad (S17)$$

$$N_{Avog} = 6.022 \times 10^{23}\ mol^{-1} \quad (S18)$$

$$n(AgMNLC) = N(AgMNLC) / N_{Avog} = 9.20 \times 10^{-9}\ mol \quad (S19)$$

7) How many mol of Ag are available in the second step of Au-AgNSs formation?

$$V(AgNO_3) = 12.5\ mL \quad (S20)$$

$$c(AgNO_3) = 0.2\ mM \quad (S21)$$

$$n(AgNO_3) = 2.50 \times 10^{-6}\ mol \quad (S22)$$

8) Abundance of Ag added into the reaction mixture in the second step of the seed-mediated growth procedure over the estimated Ag monolayer coverage (AgMNLC):

$$2.50 \times 10^{-6} / 9.20 \times 10^{-9} = 272 \quad (S23)$$

The same type of calculations can be done for AuCitr particles assuming their sizes of 50 nm in diameter and the same premises as mentioned above. Then, the abundance of Ag added into the reaction mixture in the second step of the seed-mediated growth procedure over the estimated Ag monolayer coverage (AgMNLC) is 681.

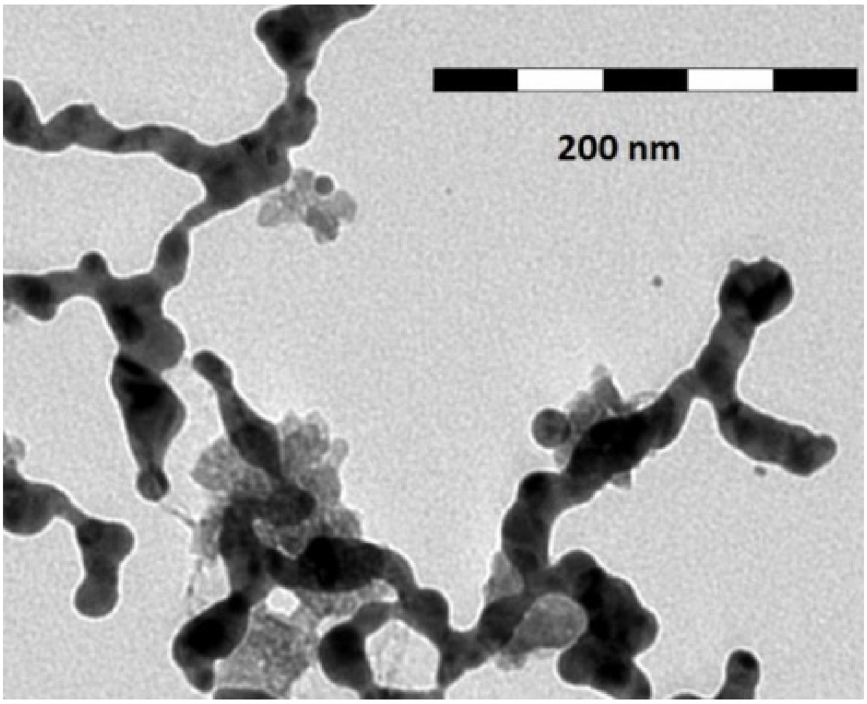

A

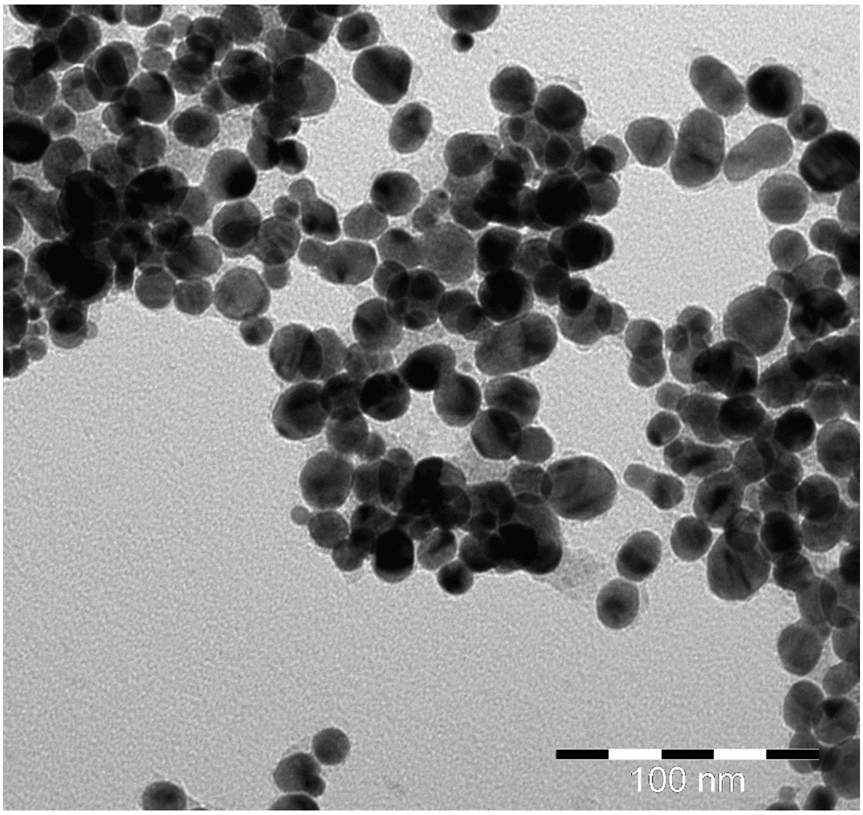

B

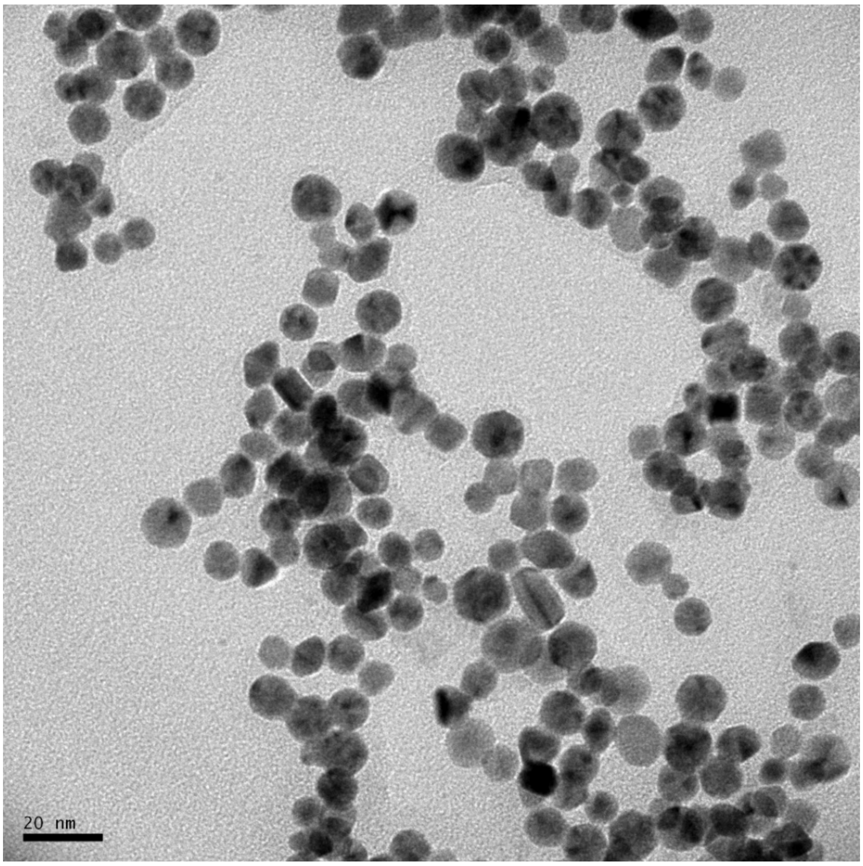

C

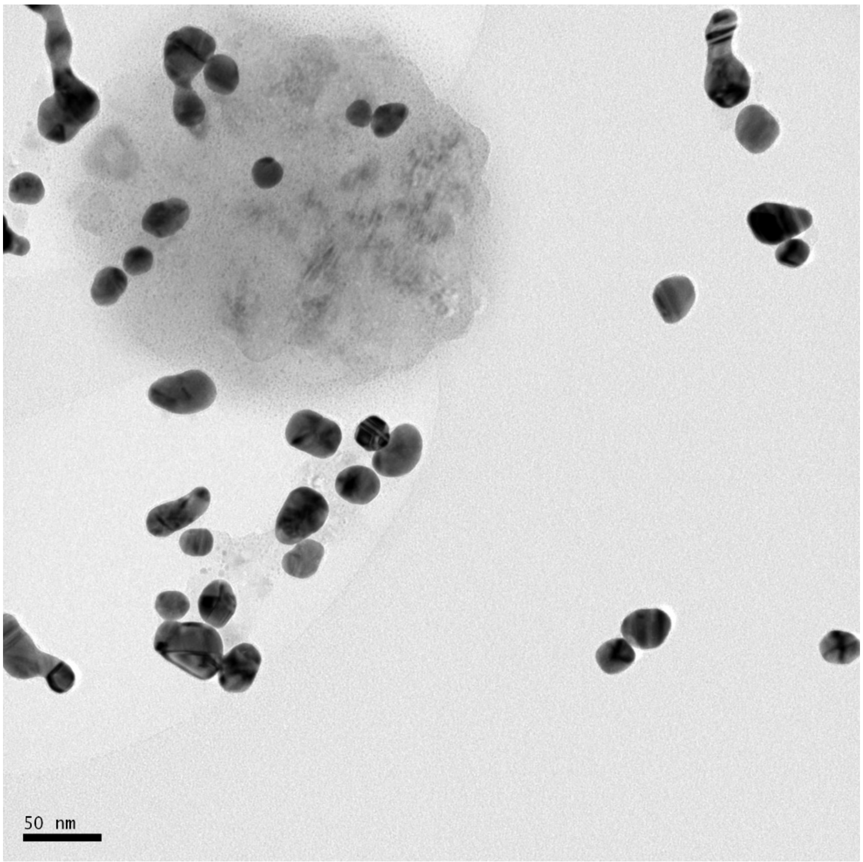

D

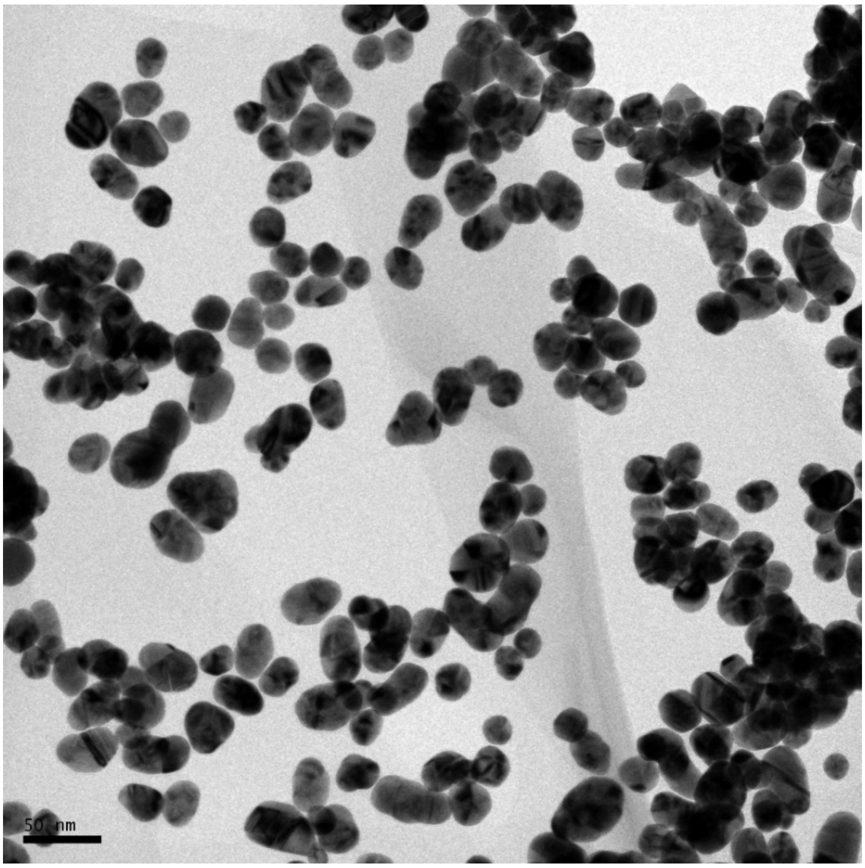

E

Figure S1. TEM images of Au seeds: (A) AuBH-fr, (B) AuBOH-fr, (C) AuBhr, (D) AuCitr-fr, (E) AuCitr.

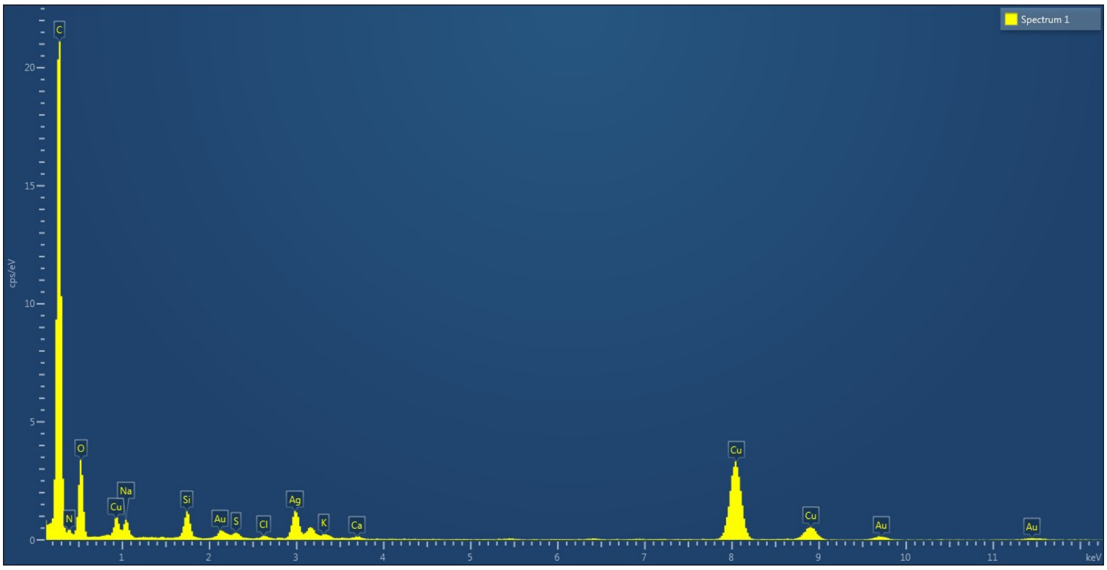

A

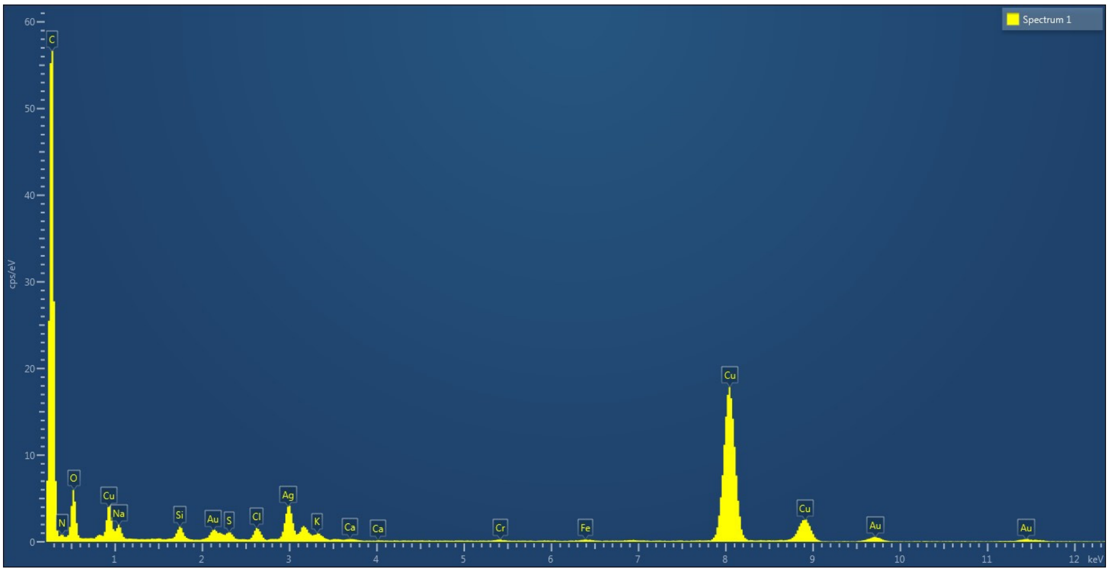

B

Figure S2. EDS (energy dispersive spectroscopic) analysis for selected TEM images of AuBhr_Ag+_Asc (A) and AuCitr_Ag+_Asc (B).

Long-term development of Au-AgNSs

The final Au-AgNSs prepared by seed-mediated growth procedure exploiting Au seeds of two different types (AuBhr and AuCitr), using either the classical (Ag+ prior to Asc) or reverse (Asc prior to Ag+) order of reactants were stored in dark at room temperature for 9 months in order to investigate their long-term development and stability. Then, DLS measurements were performed because it reflects the changes in particle size distribution of Au-AgNSs dispersed in aqueous solutions. The results are summarized in Table S1 and can be directly compared with the results shown in Table 2.

Table S1. Particle size distributions and zeta potential values of selected 9 months-aged Au-AgNSs.

**Sample code**	**Mean particle sizes and their percentual contents based on light intensity changes**				**Zeta potential values**
	[nm]	[%]	[nm]	[%]	[mV]
AuBhr_Ag+_Asc	77 ± 6	82	18 ± 1	18	-16.2 ± 1.3
AuBhr_Asc_Ag+	64 ± 2	69	13 ± 4	31	-23.4 ± 1.7
AuCitr_Ag+_Asc	55 ± 2	85	8 ± 1	15	-11.7 ± 1.6
AuCitr_Asc_Ag+	70 ± 1	79	13 ± 1	21	-10.9 ± 1.2

Bimodal particle size distribution (based on intensity changes of light scattering – Table S1) stayed valid for all four systems included in the investigation of Au-AgNSs long-term development: AuBhr_Ag+_Asc, AuBhr_Asc_Ag+, AuCitr_Ag+_Asc, AuCitr_Asc_Ag+. The percentual portion of bigger vs. smaller particles in 9-months-aged Au-AgNSs remained virtually the same as that of few-hours aged Au-AgNSs (the maximal change is of 6 %) – compare Tables S1 and 2, respectively. However, the standard deviation error of the mean size of the portion of bigger particles significantly decreased (from the value being almost half of the mean particle size to a few nanometers – compare Tables 2 and S1). This may be attributed to the finished process of particle growth after 9 months.

The growth of mean particle sizes in long period of time seems to be more pronounced in AuBhr systems (e.g. from 67± 38 nm, in Table 2, to 77 ± 6 nm, in Table 3) than in AuCitr, regardless the order of reactants. This is consistent with the generally accepted

fact that citrate-reduced AuNPs are very stable due the presence of citrate and acetone residues on NP surfaces. The importance of seeds stability in seed-mediated growth was discussed in [1]. Hence, stable Au-AgNSs can be generated from AuCitr. It was already manifested by itself (especially by the similarity of UV-Vis spectra – Figure 3B) when the order of reactants discussed in section 3.3 of this work.

Interestingly, the value of the mean particle size of the portion of bigger NPs in AuCitr_Ag+_Asc (55 ± 2 nm) aged for 9 months is much smaller than that of AuCitr_Asc_Ag+ (70 ± 1 nm) aged for 9 months, i.e. by 15 ± 2 nm – Table S1; while the opposite statement can be said about Au-AgNSs exploiting AuBhr as seeds and aged for 9 months (mean value of bigger particles in AuBhr_Ag+_Asc is about 13 ± 6 nm larger than that of AuBhr_Asc_Ag+ - Table S1). It is thus consistent with the observation made for few-hours-aged Au-AgNSs and correlates well with the assumption about Ag+ vs. Asc impact on Au seeds as schematically depicted in Scheme 1.

Considering colloidal stability of Au-AgNSs, zeta potential values need to be determined and evaluated. As it is seen in Table S1, zeta potential values of all four Au-AgNSs are negative which supports the assumption that (poly)borates, citrates, acetone residues, ascorbate and/or dehydroascorbate are present on these NSs surfaces. Au-AgNSs containing AuBhr seeds (which zeta potential manifested itself by the value of -25 ± 5 mV) revealed more negative values of zeta potential than those arisen from AuCitr seeds (which zeta potential manifested itself by two negative values: the dominant part (82%) of -26 ± 13 mV and the minor part (8%) of -15 ± 7 mV). Consequently, the Au-AgNSs containing AuCitr seeds could be more prone to aggregation than those containing AuBhr seeds when a suitable adsorbate added. Moreover, it should be kept in mind that zeta potential value is very dependent on pH of the medium which slightly differs in Au-AgNSs systems containing AuBhr and/or AuCitr as seeds (see pH values in Table 2).

Furthermore, the order of reactants as a factor influencing the final Au-AgNSs properties, here zeta potential values, is more pronounced in systems exploiting AuBhr as seeds than in the systems containing AuCitr seeds. It correlates well with the UV-Vis spectroscopic observation: while AuBhr_Ag+_Asc substantially differs from AuBhr_Asc_Ag+, both AuCitr-containing systems are very similar to each other as for their LSPR spectra (compare Figures 3A and 3B).

[1] L. M. Liz-Marzán, M. Grzelczak, Growing anisotropic crystals at the nanoscale, Science (80), 356 (2017) 1120-1121. https://doi.org/10.1126/science.aam8774

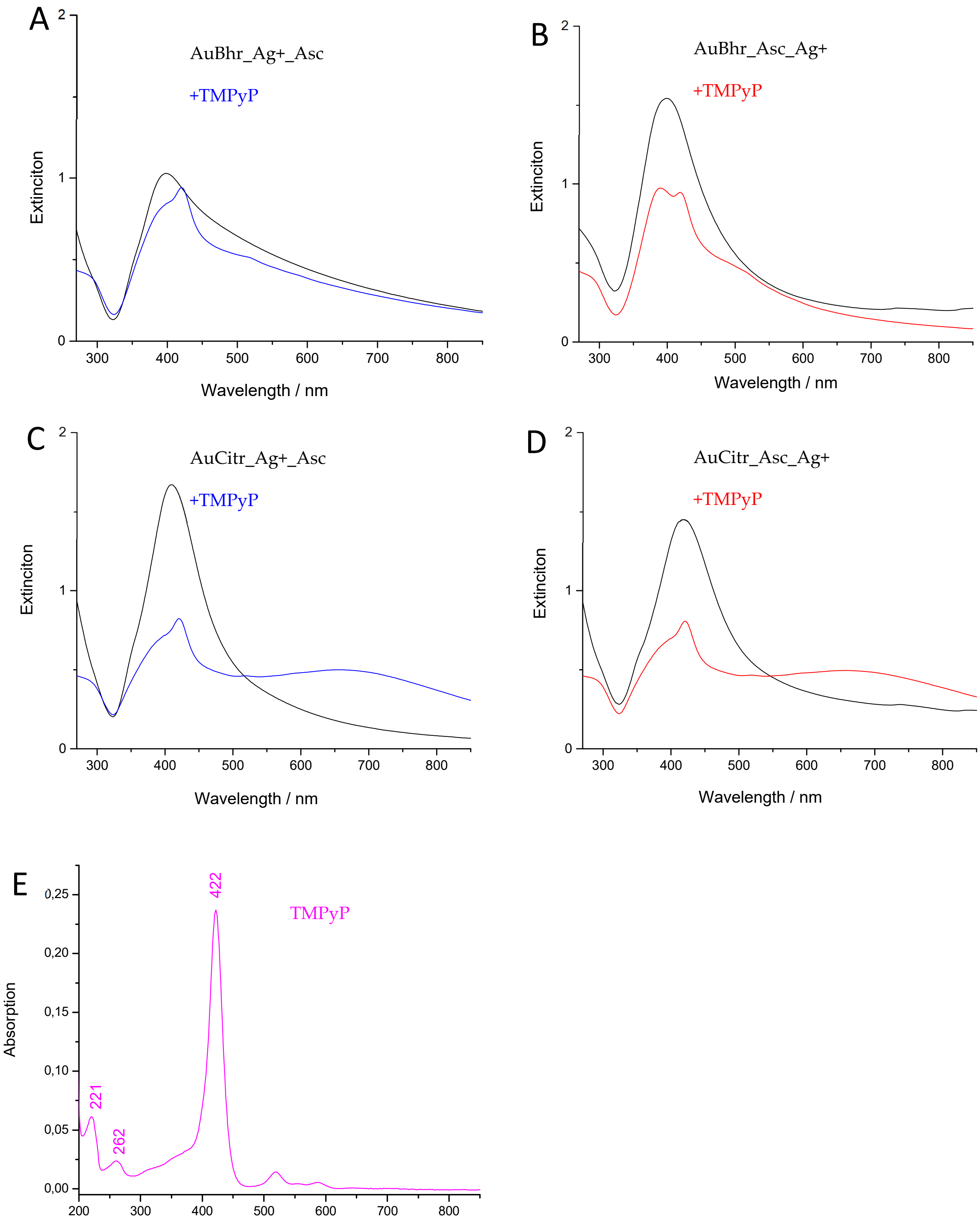
A
AuBhr_Ag+_Asc
+TMPyP
Extinciton
Wavelength / nm
B
AuBhr_Asc_Ag+
+TMPyP
Extinciton
Wavelength / nm
C
AuCitr_Ag+_Asc
+TMPyP
Extinciton
Wavelength / nm
D
AuCitr_Asc_Ag+
+TMPyP
Extinciton
Wavelength / nm
E
TMPyP
422
221
262
Absorption

Figure S3. Extinction spectra of each Au-AgNSs measured prior (black curves) and after TMPyP addition (colored curves) for (A) AuBhr_Ag+_Asc, (B) AuBhr_Asc_Ag+, (C) AuCitr_Ag+_Asc, (D) AuCitr_Asc_Ag+, and (E) absorption spectrum of TMPyP in 1×10^{-6} M concentration in the final systems.

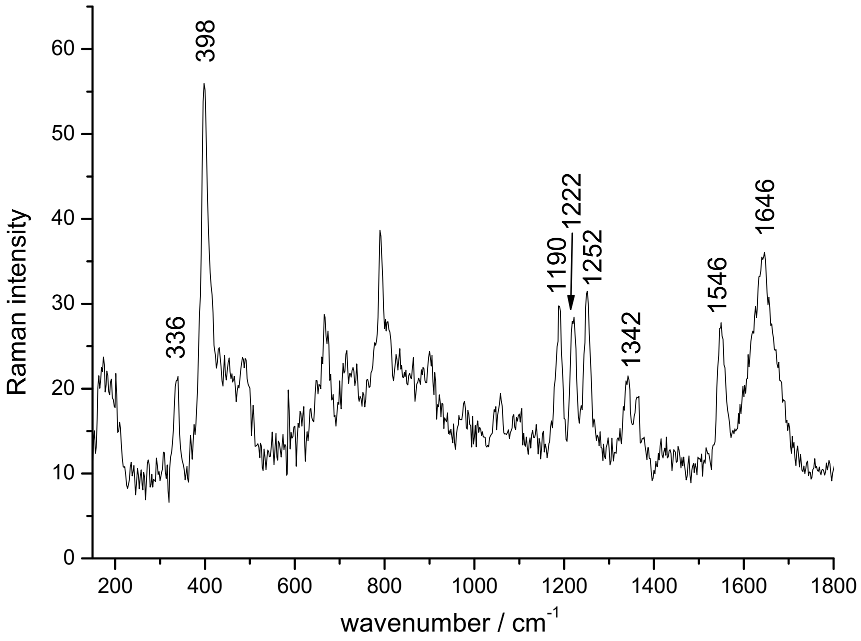

Figure S4. SERS spectrum of TMPyP (1×10^{-6} M) adsorbed on AgNPs prepared by borohydride reduction (AgBhr) and aged for 9 months.

Experimental details for AgBhr colloid preparation (aged for 9 month and used with TMPyP as a reference system): 3.43 mg $NaBH_4$ was dissolved in 75 mL of deionized water submerged in an ice bath. The mixture was stirred at the rate of 1000 rpm while adding drop-wise 9 mL of 2.2 mM $AgNO_3$ in the middle of the stirring vortex. The mixture was withdrawn from an ice bath after 6 minutes and subsequently stirred for 1 hour until reaching room temperature. The AgBhr colloid was then stored in dark at room temperature for 9 months and used with TMPyP as a reference system of Au-AgNSs in SERS measurements.

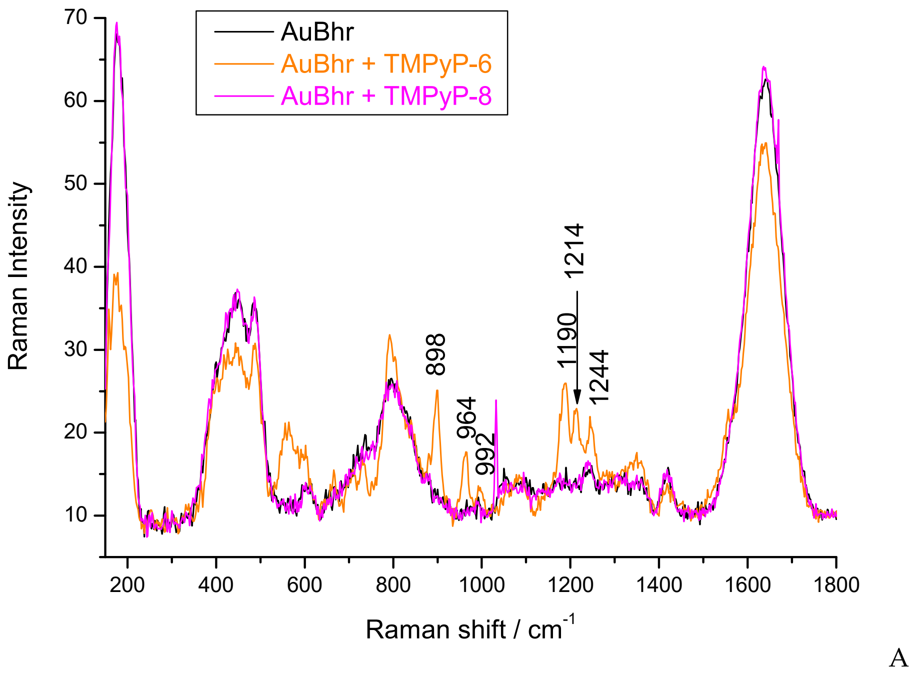

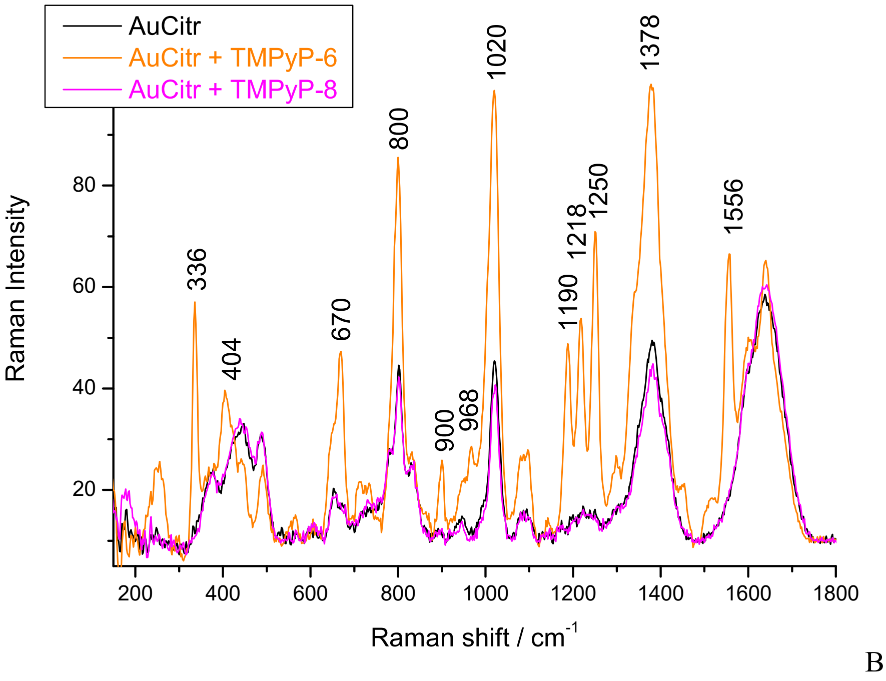

Figure S5. Overlaid SERS spectra of TMPyP (1×10^{-6} M in orange and 1×10^{-8} M in magenta) adsorbed on Au seeds reduced by: (A) borohydride, (B) citrate. Spectra of Au seeds without TMPyP addition (black curves) are shown for the sake of direct comparison. SERS spectra were recorded by using 785 nm excitation laser line, 300 mW, 2s 30x, and automatic baseline corrected.

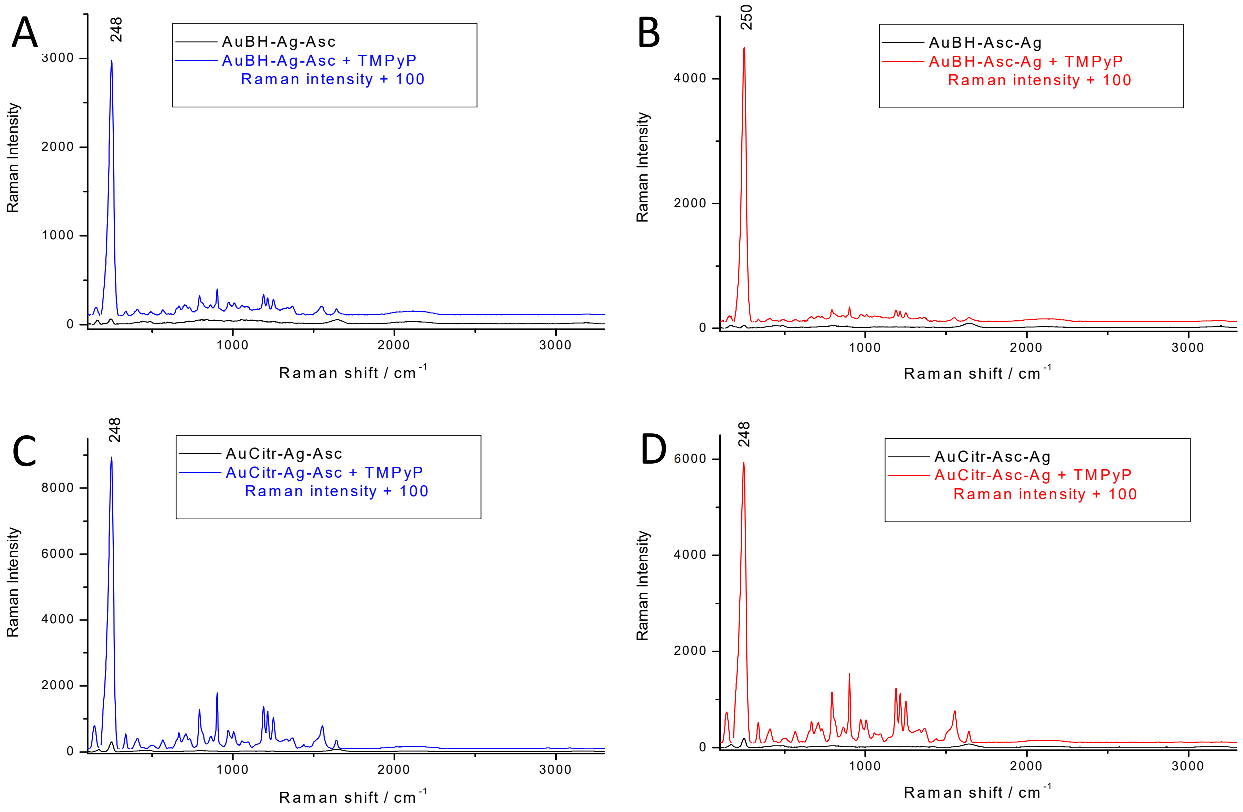

Figure S6. Whole intensity range of SERS spectra of TMPyP (final concentration of 1 × 10^{-8} M) adsorbed on: (A) AuBhr_Ag+_Asc, (B) AuBhr_Asc_Ag+, (C) AuCitr_Ag+_Asc, (D) AuCitr_Asc_Ag+.

Calculation of the monolayer coverage TMPyP concentration on the surface of Au-AgNSs prepared by seed-mediated growth:

Theoretically there are 2.87 × 10^{11} pieces of AuBhr particles (whereas 1.83 × 10^{10} pieces of AuCitr particles) serving as seeds in the second step of Au-AgNSs generation. Under the assumptions that Ag atoms are deposited only on Au particle surfaces, the final Au-AgNSs are isolated spheres with the mean diameter of 70 nm, and all added Au(III) and Ag(I) ions are consumed into Au-AgNSs in the process of their formation, the available surface area of the final Au-AgNSs can be estimated as follows:

- The surface area of a spherical Au-AgNS of 70 nm in diameter:

$$S(\text{Au-AgNSs}) = 4 * \Pi * R^2 \quad \text{(S24)}$$

$$R = 35 \text{ nm} \quad \text{(S25)}$$

$$S(\text{Au-AgNSs}) = 4 * \Pi * 35^2 \text{ nm}^2 = 15{,}393.80 \text{ nm}^2 \quad \text{(S26)}$$

- Available surface area of all Au-AgNSs present in 1 mL which is used in SERS measurements:

$$V(\text{final Au-AgNSs}) = 13.25 \text{ mL} \quad \text{(S27)}$$

$$\text{Number of Au-AgNSs in 1 mL….N(Au-AgNS-1mL)} = 2.87 \times 10^{11} / 13.25 = 2.17 \times 10^{10} \quad \text{(S28)}$$

$$\mathbf{S(Au\text{-}AgNSs\text{-}1mL)} = S(\text{Au-AgNSs}) * N(\text{Au-AgNSs-1mL}) = \mathbf{33{,}404.55 \times 10^{10}\ nm^2} \quad \text{(S29)}$$

- The surface area of a porphyrin molecule:

Based on the assumption made in [Sloufova-Srnova I., Vlckova B., NanoLett 2002, 2, 121-125], the porphyrin diagonal dimension is ~2 nm. Therefore, the size of a porphyrin site (labeled as a) can be calculated according to Pythagorean theorem:

$$a = (1^2 + 1^2)^{1/2} \tag{S30}$$

Then, the surface area of a porphyrin molecule can be estimated:

$$S(\text{porphyrin}) = a^2 = (2^{1/2})^2\ \text{nm}^2 = 2\ \text{nm}^2 \tag{S31}$$

- Number of porphyrin molecules adsorbed on the available surface area of all Au-AgNSs in 1mL (This calculation is based on the assumption that porphyrins are adsorbed next to each other creating thus a monolayer on Au-AgNSs):

$$N(\text{porphyrins in 1 mL}) = S(\text{Au-AgNSs-1mL}) / S(\text{porphyrin}) = 33{,}404.55 \times 10^{10} / 2 = 16{,}702.27 \times 10^{10} \tag{S32}$$

- The porphyrin concentration corresponding to the monolayer coverage…c(MNLC TMPyP):

$$n(\text{porphyrins in 1 mL}) = N(\text{porphyrins in 1 mL}) / N_{Avog} \tag{S33}$$

$$c(\text{MNLC TMPyP}) = n(\text{porphyrins in 1 mL}) / V \tag{S34}$$

$$V = 1\ \text{mL} = 1 \times 10^{-3}\ \text{L} \tag{S35}$$

$$c(\text{MNLC TMPyP}) = 16{,}702.27 \times 10^{10} / 6.022 \times 10^{23} / 1 \times 10^{-3}\ \text{M} = \mathbf{2.77 \times 10^{-7}\ M} \tag{S36}$$
